# Connecting Patient and Provider Burnout to Eye Exam Frequency among Latinx Older Adults with Diabetes Mellitus

**DOI:** 10.1093/geroni/igab046.2383

**Published:** 2021-12-17

**Authors:** Adrienne Martinez-Hollingsworth, Theodore Friedman, Mohsen Bazargan

**Affiliations:** 1 Samuel Merritt University, Los Angeles, California, United States; 2 Charles R. Drew University of Medicine and Science, Los Angeles, California, United States

## Abstract

Among Latinx older adults, our current understanding of barriers to eye exam often fails to consider the impact of patient and provider burnout which can decrease treatment adherence and recommendation receptivity in this group. The purpose of this study was to examine correlates of eye exam frequency among Latinx older adults in South Los Angeles and explore associations reflecting patient and/or provider burnout. Data analysis was informed by the Secret Self-Management Loop and the Burnout Dyad conceptual models. This secondary analysis used data collected from a convenience sample of non-institutionalized Latinx adults 55+ in South LA (n=165) and used multinomial regression analysis. Outcome variable is recency of eye exam, independent variables are self-reported health, including diabetes mellitus diagnosis, and either patient or provider burnout (that are functions of grouped demographic or quality of care variables). Variables associated with Provider Burnout, appear to represent a larger influence on eye examination frequency then variables associated with Patient Burnout, with the most influential factor being provider recommendation. A surprising finding was the number of participants who had never received this recommendation from a provider (21%). One-third (32%) of participants with diabetes mellitus had not had an eye examination within 12 months and almost one-fifth (13%) of participants with diabetes who had received this recommendation had not received the exam. Further exploration is needed to support a better understanding of how both patient and provider burnout impacts adherence to eye examination and other preventive care recommendations for diabetes mellitus among Latinx older adults.

